# The efficacy and safety of allogeneic stem cell transplantation in Mevalonate Kinase Deficiency

**DOI:** 10.1186/s12969-022-00716-4

**Published:** 2022-07-29

**Authors:** Jerold Jeyaratnam, Maura Faraci, Andrew R. Gennery, Katarzyna Drabko, Mattia Algeri, Akira Morimoto, Tiarlan Sirait, Arjan C. Lankester, Michael Albert, Benedicte Neven, Joost Frenkel, on behalf of the EBMT Inborn Errors Working Party

**Affiliations:** 1 Department of Pediatrics, University Medical Center Utrecht, 3584 EA Utrecht, the Netherlands; 2 Department of Hematology-Oncology, Stem Cell Transplantation, IRCCS Istituto G. Gaslini, Genoa, Italy; 3 Translational and Clinical Research Institute, Newcastle University, and Paediatric Haematopoietic Stem Cell Transplant Unit, Great North Children’s Hospital, Newcastle upon Tyne, UK; 4 Department of Pediatric Hematology, Oncology and Stem Cell Transplantation, Medical University of Lublin, Lublin, Poland; 5 Department of Pediatric Hematology and Oncology and Cell and Gene Therapy, IRCCS, Ospedale Pediatrico Bambino Gesù, Rome, Italy; 6 Department of Pediatrics, Jichi Medical University, Shimotsuke, Japan; 7 EBMT Data Office, Leiden, The Netherlands; 8 Department of Pediatrics, Division of Immuno-Hematology and Stem Cell Transplantation, Leiden University Medical Center, Leiden, The Netherlands; 9 Department of Pediatrics, Dr. von Hauner Children’s Hospital, University Hospital, LMU, Munich, Germany; 10 Unité d’Immuno-hematologie et Rhumatologie Pédiatrique, Hôpital Necker-Enfants Malades, assistance publique hopitaux de paris, Paris, France; Université de Paris, Paris, France

**Keywords:** Inborn metabolic disease, Mevalonic aciduria, Therapeutic options, Auto-inflammation, Allogeneic stem cell transplantation

## Abstract

**Objectives:**

Mevalonate kinase deficiency (MKD) is a rare autoinflammatory syndrome. Several reports have described allogeneic hematopoietic stem cell transplantation in severely affected patients, sometimes with promising results. In view of the scarcity of data, this study aims to analyse the efficacy and safety of allogeneic hematopoietic stem cell transplantation (HSCT) to give a more complete overview of this treatment.

**Methods:**

This multicentre retrospective study on behalf of the European Society for Blood and Marrow Transplantation aimed to include all MKD patients who had undergone allogeneic HSCT. All centres related to EMBT and centres that have reported cases of allogeneic HSCT in the literature were contacted via the EBMT data office.

**Results:**

We analyzed 9 patients (5 male). Treosulfan based conditioning was the most frequently used conditioning regimen. Engraftment occurred in all but one patient. Source of stem cells was cord blood (*n* = 2), peripheral blood stem cells (*n* = 4) and bone marrow (*n* = 5). Two patients needed a second transplantation due to an incomplete response or primary graft failure. Seven patients went into complete remission after stem cell transplantation. At final follow-up these patients reported no symptoms of MKD.

Four patients suffered from grade II-IV acute graft-versus-host disease (GvHD). During follow-up two patients died due to transplantation related complications.

**Conclusion:**

In conclusion, allogeneic stem cell transplantation represents an effective treatment for the most severely affected MKD patients. However, treatment-related morbidity and mortality are significant. Transplantation may be justified in patients with a severe disease course on conservative therapy.

## Introduction

Mevalonate kinase deficiency (MKD) is a rare autosomal recessive autoinflammatory syndrome characterized by fever and generalized inflammation. The disease has a broad clinical spectrum in manifestations and severity. Typically, it presents as two clinical phenotypes, known as the Hyper Immunoglobulinemia D and periodic fever syndrome (HIDS) and Mevalonic Aciduria (MA) with overlap between the two. Most MKD patients experience fever, gastrointestinal symptoms, lymphadenopathy, arthralgia, myalgia, skin rash and mucosal ulcers. Furthermore, patients with the more severe Mevalonic Aciduria phenotype can also exhibit dysmorphic features, pre- and postnatal growth retardation, neurological and ocular involvement [1–3].

MKD is caused by mutations in the *mevalonate kinase* gene (*MVK*), that encodes mevalonate kinase, an enzyme which is part of the isoprenoid biosynthesis pathway. Mutations in this gene lead to reduced mevalonate kinase activity varying from 0.5 to 28% as compared to that of healthy controls, the lowest being found in the most severe phenotype Mevalonic Aciduria. This leads to accumulation of mevalonic acid, which can lead to elevated amounts in urine samples. The diagnosis can be confirmed by the presence of two pathogenic *MVK* mutations, and/or by measurement of reduced mevalonate kinase activity in fibroblasts or leukocytes [4].

Treatment of these patients is largely individualized, although treatment with IL-1 blockers (canakinumab, anakinra) has proven to be effective in a substantial proportion of patients [5–7]. Other therapies can include non-steroidal anti-inflammatory drugs (NSAIDs), corticosteroids and the TNF-α blocking agent etanercept [5, 8]. However, a small group of severely affected patients do not respond sufficiently to these treatments. In other cases, the fever episodes can be controlled, but the neurological and ocular decline cannot be prevented. The inflammatory phenotype of MKD is mainly caused by the lack of mevalonate kinase in phagocytes [9]. Therefore, replacing these cells by genetically normal cells might be curative. Several reports have described allogeneic hematopoietic stem cell transplantation (HSCT) in severely affected patients, sometimes with promising results [10–12]. In view of the scarcity of data, this study aims to evaluate the efficacy and safety of allogeneic HSCT in a larger multi-center cohort.

## Methods

This multicenter retrospective study conducted on behalf of Inborn Errors Working Party of the The European Society for Blood and Marrow Transplantation (EBMT) (study number 8427021) aimed to include all known MKD patients who had undergone allogeneic HSCT. All centers that had reported patients with allogeneic into the EBMT registry and in the literature were contacted via the EBMT data office. A pseudonymized data collection questionnaire was sent to participating centers to obtain required information missing from the registry. Ethical approval was obtained according to EBMT guidelines.

### Endpoints

The primary endpoint of this study was to evaluate the efficacy and safety of allogeneic HSCT in MKD. Data about engraftment and chimerism at different timepoints, the number of flares and the clinical symptoms occurring before and after transplantation as well as the clinical and neurological status at last follow up were collected. Overall survival, cause of death, incidence and severity of acute and chronic Graft-versus-Host Disease (GvHD) incidence of other morbidities related to HSCT were also retrieved. Acute and chronic GvHD were graded according to modified Seattle criteria and NIH consensus standards where available [13, 14].

### Statistics

Categorical variables are presented as frequencies and percentages. Numeric variables are presented as the median and range.

## Results

In the EBMT registry (> 600 centers) 7 patients from 5 centers could be identified. Two additional patients could be included by contacting centers from published case reports. The final analysis comprises 9 MKD patients from 6 centers, who underwent 11 transplantations. Five male patients were included. The median age at transplantation was 2.6 years (range 0.4-8.3 years). The disease course started during the neonatal period in all but one. Four patients were born prematurely. The median number of fever episodes was 24 per year (range 4-continuous inflammation) and the median duration of episodes was 5 days (range 3-7 days). Most patients experienced typical MKD symptoms, such as abdominal pain, vomiting, diarrhea, lymphadenopathy, maculopapular rash. In addition, several patients also suffered from severe features, including dysmorphic features, hypotonia, psychomotor retardation, oculomotory apraxia, macrophage activation syndrome and AA-amyloidosis. One patient suffered from hydrocephalus. The clinical features before HSCT are summarized in Table 1. MVK-enzyme activity was < 1% compared to healthy controls when measured (*n* = 3) and urine mevalonic acid was markedly elevated in the 3 assessed patients. All but one patient had been treated with biologicals (anakinra, etanercept, canakinumab).

**Table 1:** Baseline demographic and disease characteristics

Characteristics	Patients (***n*** = 9)	
Male, n (%)	5	(56%)
Age at onset		
-neonatal period	8	(89%)
Median number of flares per year	24	(4-continuous)
Median duration of flares in days	5	(3-7 days)
Symptoms		
-Abdominal pain	6	(67%)
-Diarrhea	5	(56%)
-Vomiting	6	(67%)
-Lymphadenopathy	4	(44%)
-Maculopapular rash	5	(56%)
-Arthralgia	4	(44%)
-Arthritis	1	(11%)
-Hepatosplenomegaly	4	(44%)
-Malaise	5	(56%)
-Psychomotor retardation/ataxia	5	(56%)
-Hypotonia	3	(33%)
-Hypospodia	1	(11%)
-Oculomotory apraxia	1	(11%)
-Macrophage activation syndrome	1	(11%)
-AA-amyloidosis	1	(11%)
Previous treatments		
-NSAIDs	5	(56%)
-Corticosteroids	7	(78%)
-Anakinra	7	(78%)
-Etanercept	1	(11%)
-Canakinumab	2	(22%)

Most patients underwent transplantation because of active disease, refractory to biologicals. Patient 1 (Table 2) presented at birth with hemophagocytic lymphohystiocytosis (HLH) Generalized edema, skin eruption and hepatosplenomegaly were noticed at birth. He had anemia and low platelet count, elevation of lactate dehydrogenase enzyme (LDH), direct bilirubin, ferritin and soluble interleukin-2 receptor (6.440 U/ml), and low fibrinogen (109 mg/dl). Cytotoxic activity was moderately reduced (30% of normal value). These findings fulfilled diagnostic criteria of HLH though no known genetic abnormality was detected. He was treated according to the HLH-2004 protocol, did not attain remission and underwent allogeneic HSCT. The diagnosis of MKD was made retrospectively after transplantation. This patient had a relevant family history. His oldest sister died in utero at a gestational age of 23 weeks due to hydrops fetalis and hepatosplenomegaly. The second oldest sister was born at 27 weeks of gestation with hepatosplenomegaly, thrombocytopenia and anemia, and died of respiratory failure.

**Table 2:** Detailed information of all patients before allogeneic stem cell transplantation

Patient	Gender	Age at onset	Mutations	Severe manifestations before HSCT	Previous treatments	Reason for stem cell transplantation
1	Male	Neonatal period	p.A147T+ c.227-1G > A	Psychomotor retardation, hydrocephalus, hypospadias, inguinal hernia	Corticosteroids	Initial diagnosis of HLH
2	Male	Neonatal period	p.G326R + p.G326R	Mild ataxia, hepatosplenomegaly	Corticosteroids, etanercept,anakinra	active disease refractory to biologicals
3	Male	Neonatal period	p.V8M + p.V8M	mild psychomotor delay, severe hypotonia, perimalleolar cellulitis, hepatosplenomegaly, microcytic anemia,failure to thrive, atypical craniofacial dysmorphic features	Anakinra	active disease refractory to biologicals
4	Female	Neonatal period	p.T237S + p.T237S	axial hypotonia and motor retardation	Anakinra	active disease refractory to biologicals
5	Male	Neonatal period	p.R124K + p.L2971	hypotonia and oculomotory apraxia, hepatomegaly, macrophage activation syndrome, AA-amyloidosis	Corticosteroids, Anakinra canakinumab	active disease refractory to biologicals
6	Female	Neonatal period	p.R215X + p.V377I		Corticosteroids, anakinra canakinumab, tocilizumab, adalimumab	active disease refractory to biologicals
7	Female	Neonatal period	p.R388X + second mutation unknown	Hepatosplenomegaly	Corticosteroids, anakinra	active disease refractory to biologicals
8	Female	Neonatal period	p.1268 T- p.V310M		Corticosteroids, anakinra	active disease refractory to biologicals
9	Male	3 years	p.V377I + p.G211A		Corticosterioids	active disease refractory to biologicals, rising serum amyloid A

### Transplantation

All data regarding transplantation procedure and outcome are summarized in Table 3.

**Table 3:** Detailed information of all patients regarding allogeneic stem cell transplantation and outcome

P	Age at HSCT (yrs)	Conditioning	Donor type	Graft source	HLA match	Acute GvHD	Follow-up period after SCT (months)	Chimerism in myeloid cells at last follow-up (method)	neurological manifestations at last follow-up	Normal schooling	Normal growth	Outcome
1	1.1	ALG, cy, VP16, flu, mel.	MUD	CB	8/8	Grade II (skin/gut)	1	–	hydrocephalus	NA	NA	Death
2	2.6	Cy, busulfan	MSD	BM	10/10	(−)	194	100% (FISH)	No	yes	low weight	Alive
3	2.9	Cy, busulfan ATG	MUD	CB	8/12	Grade II (skin)	149	100% (STR)	PRES. Seizures ^a^	yes	yes	Alive
4	0.7	treo, flu, TT alfa/beta/Cd19 depletion	MMRD (haplo)	PBSC	6/12	(−)	307	100% (STR)	No	yes	yes	Alive
5	1.8	treo, flu, TT, alfa/beta/Cd19 depletion	MMRD (haplo)	PBSC	6/12	(−)		Graft failure				
	2.0	busulfan, flu, alemtuzumab, alfa/beta/Cd19 depletion	MMRD	PBSC	6/12	(−)	16	100% (STR)	No	yes	yes	Alive
6	6.6	treo, flu, TT ATG, rituximab	MMRD (haplo)	PBSC	6/12	(−)	60	100% (STR)	No	yes	yes	Alive
7	0.4	treo, flu	MSD	BM		(−)		43%inBM		NA^b^		Alive
	2	busulfan, flu, ATG	MUD	BM	10/10	–	38	100% (STR)	No	NA	yes	Alive
8	1.7	treo, flu, TT, ATG	MMUD	BM	9/10	Grade IV (skin/liver)	3	–	No	NA	NA	Death
9	8.3	busulfan, cy	MSD	BM	12/12	Grade IV (skin/gut)	127	100% STR	No	yes, now working	yes	Alive

Legend: Conditioning: *ATG* Anti-thymocycte globulin, *ALG* Anti-lymphocyte globulin, *cy* Cyclophosphamide, *VP16* Etoposide, *flu* Fludarabine, *mel* Melphalan, *treo* Treosulfan

donor type: *MUD* Matched unrelated donor, *MSD* Mismatched sibling donor, *MMUD* Mismatched unrelated donor, *MMRD* Mismatched related donor

Graft source: *CB* Cord blood, *BM* Bone marrow, *PBSC* Peripheral blood stem cells

^a^The patient developed posterior reversible encephalopathy syndrome, which led to seizures requiring anti-epileptic therapy

^b^: not applicable, the patient will attend school after being completely vaccinated

Treosulfan-based conditioning was the most frequently used conditioning regimen (*n* = 5). Serotherapy (antithymocyte or antilymphocyte globulin) was used in 4 cases (Table 3). Source of stem cells was cord blood (*n* = 2), peripheral blood stem cells (PBSC) (*n* = 4) and bone marrow (*n* = 5).

Stem cells came from a matched sibling donor (MSD) in 3 occasions, a matched unrelated donor (*n* = 3), a mismatched unrelated donor (MMUD) and, 4 mismatched related donors (MMRD) (Table 3). The haploidentical transplants in patient 4 and 5 were performed using the platform with alfa/beta/CD19 depletion of PBSC collected from the parent.

Engraftment (absolute neutrophil count ≥0.5 × 10^9^/L) occurred in all patients except patient 5 after a median of 20 (range 6 – 31) days. A platelet count ≥50 × 10^9^/L was reached by 6 patients after a median of 23.5 (18 – 61) days.

Donor chimerism results are summarized in Table 2. All seven surviving patients reached full donor myeloid chimerism. Two patients reached full chimerism after a second transplant. Two patients died due to transplant-related complications without achieving full donor myeloid chimerism (patient 1 and 8).

### Efficacy of allogeneic hematopoietic stem cell transplantation

Detailed information about the post-transplant course is listed in Table 3. All but one patient had resolution of inflammatory symptoms after HSCT. At last follow-up these patients reported no symptoms of MKD and, six patients did not exhibit severe neurological manifestations. Most patients attended regular school. However, one patient (patient 7) who had reached 43% chimerism after an HSCT from her brother still reported some symptoms after transplantation, namely maculopapular rash. Her brother was heterozygous for the mutation (p.R388X). During follow-up she had an MVK-enzyme activity of around 5% compared to healthy controls, probably due to the combination of limited engraftment and the donor cells carrying the defect. This patient underwent a second allogeneic HSCT from a MUD 1.5 years after the first transplantation, which led to 100% chimerism in myeloid cells and clinical remission. Another patient (patient 5) required a second transplantation due to primary graft failure from day 26 after HSCT. He received a second transplantation from the same donor (his father) and achieved complete remission afterwards.

MVK-enzyme activity was not measured after transplantation in the other patients.

### Safety of allogeneic hematopoietic stem cell transplantation (transplant related morbidity and mortality)

Four patients suffered from acute GvHD (aGvHD) varying from grade II to grade IV. The median time of onset was 0.5 months after transplantation.

Two patients with grade II aGvHD had involvement of the skin and/or gastro-intestinal tract. Grade IV aGvHD was reported in two patients with involvement of skin/liver and skin/gut.

Limited chronic graft versus host disease occurred in one patient (patient 4) 3.4 months after HSCT. This was a first episode and resolved over time. Patient 9 also experienced limited chronic GvHD which involved the skin requiring oral tacrolimus and topical corticosteroids.

Patient 3 developed posterior reversible encephalopathy syndrome (PRES). This led to mesial temporal sclerosis, which led to seizures requiring anti-epileptic therapy.

During follow-up two patients died of transplant-related causes. One (patient 8) died 3.5 months after transplantation due to multiorgan failure which was ascribed to uncontrolled GvHD. Admission to the IC unit was required because of respiratory failure, followed by kidney failure and circulatory failure. This was complicated by infections due to *Enterococcus faecium* and *Candida albicans*.

The other patient (patient 1) died 1.0 months after transplantation due to progressive respiratory failure from day 6. Both patients reached engraftment before they died.

## Discussion

This report shows the results of allogeneic stem cell transplantation in MKD. Stem cell transplantation has rarely been performed for this condition. Although this cohort is small, it is able to deliver a general understanding of the safety and efficacy of this treatment in the most severely affected MKD patients.

Stem cell transplantation appears to be an effective therapy to achieve long-term remission in severe refractory MKD, with seven patients being symptom-free during follow-up. These seven patients were full donor chimeras and did not require any anti-inflammatory treatment. This is in line with the results of the case reports that have described this treatment in MKD. Six patients in this cohort have been reported in previous case reports, while three patients (patients 6, 7 and 8) had not been described yet [10, 11, 15–17]. Although some patients have been described before, the follow-up in this study is far longer than in these case reports.

Importantly, neurological improvement was suggested in a number of cases. Whether this is due to reduced pathogenic activity of stem cell-derived phagocytes in the central nervous system or that the transplanted cells provide some degree of enzyme replacement therapy benefitting the nervous system remains uncertain. The only patient with minimal residual disease activity had only one known mutation and received a transplant from a brother who was heterozygous for the same mutation. This patient underwent a second transplant from a MUD with full donor chimerism and achieved clinical remission. In order to ensure sufficient correction of the defect, it seems prudent to only use donors with fully normal MVK activity.

Transplant related morbidity and mortality remain a serious concern in a disease that can be adequately controlled by conservative measures in most affected individuals. HSCT should be reserved for the most severe MKD patients, refractory to anti-inflammatory therapies. Early identification and timely transplantation of these patients seems important to prevent patients from developing disease related sequelae or risk factors for poor HSCT outcome.

Notably, one of the patients who died had poor prognostic features, since he suffered from active HLH at the time of transplantation [18]. Although mortality was significant, early identification and timely transplantation seems important to prevent patients from developing complications. The haploidentical transplant with the alfa/beta/Cd19 depletion might be a serious therapeutic approach, since the incidence of the most frequent complications, acute and chronic GvHD, is reduced. Also of note, severe GVHD was only observed in patients with HLA-mismatched donors.

Allogeneic HSCT has been performed in other auto-inflammatory diseases and the results seem to be promising. The first report described a girl with FMF and congenital dyserythropoietic anemia (CDA). She underwent allogeneic stem cell transplantation to treat the CDA. As a side-effect her FMF went in remission and she could be weaned of all immunosuppressants [19]. Since then, many other reports have been published. Good results of allogeneic HSCT have been observed in 14 patients with deficiency of adenosine deaminase type 2 (DADA2). All patients were in complete remission during follow-up (5 months - 13 years). No mortality was reported in this cohort [20]. Allogeneic stem cell transplantation has also been described in a case of Cryopyrin-associated periodic syndrome (CAPS) who underwent allogeneic stem cell transplantation for acute lymphoblastic leukemia (ALL). During follow-up (7 years) she was in remission regarding both ALL and CAPS [21]. HSCT has been reported in 5 patients with PSTPIP1-associated myeloid-related proteinemia inflammatory (PAMI) syndrome. At a median follow-up of 2.2 years all patients had predominant or complete donor chimerism and were free of any PAMI symptoms [22]. Two patients with A20 haploinsufficiency who underwent HSCT have been described with good efficacy regarding disease activity. However, both patients already had significant irreversible damage before transplantation [23].

This study comes with a number of limitations. The small number of patients is the biggest limitation and hampers statistical analysis. However, this small number reflects the rare requirement of allogeneic HSCT in the orphan disease MKD. Further, the retrospective design has led to missing values. The EBMT collected data at stem cell transplantation centers, while many patients may have been cared for by pediatric rheumatologists or metabolic disease specialists both before and after transplantation. The large number of medical specialties and specialists involved over time in the treatment of such complex patients complicates complete data collection. Therefore, the coverage of our study cannot be ascertained.

A search through the literature yielded three additional cases that could not be included in the present study. A girl who was compound heterozygous for p.I268T/p.V310M with severe multi-organ involvement, including cholestatic liver disease, renal tubular acidosis and growth failure, first underwent a liver transplantation due to liver failure. Although the liver function improved, she kept suffering from febrile episodes, growth failure, spastic diplegia and was not able to ambulate. She underwent HSCT from an unrelated 8/8 HLA identical donor. Engraftment occurred on day 10 after transplantation and chimerism was 99% in all cell lineages at day 30. Her febrile episodes ceased and she showed substantial neurological improvement. At last follow-up 2.5 years after she followed normal schooling and showed normal no signs of spasticity or cerebellar dysfunction [24].

Another case report reported a patient who was homozygous for p.G336S. This patient had a massive hepatosplenomegaly and suffered from ascites, which required recurrent paracentesis. Despite maximal conservative therapy including prednisolone and canakinumab, the ascites persisted leading to respiratory distress. The patient underwent HSCT from an HLA-identical sister. After transplantation the hepatosplenomegaly persisted, while the ascites regressed. The patient died due to septicemia 3.5 months after transplantation [12]. The last case report described a girl born at 29 weeks gestation with anemia, thrombocytopenia and direct hyperbilirubinemia. A peritoneal drain placement was required due to massive ascites. Genetic analysis revealed a homozygous Leu297Ile mutation. Since inflammatory episodes requiring frequent hospitalizations persisted, despite prednisolone and anakinra, HSCT was performed from a 5/6 HLA matched donor. Three months post-transplant chimerism in her CD3+ lymphoid fraction was 100 and 95% in her CD15+ myeloid fraction. Urinary mevalonic levels became normal. However, 18 months post-transplant she presented with fever, arthritis and rash. Bone-marrow aspirate showed 97% chimerism of donor cells. Based on these findings, she was diagnosed with a relapse of disease. Treatment with canakinumab and oral steroids were required to control the disease [25].

In conclusion, allogeneic HSCT may be an effective treatment for the most severely affected MKD patients. Failure of anti-inflammatory therapies and very severe disease course could be indicators to select patients for this treatment. Transplant related morbidity and mortality remain a serious concern. Nonetheless, timely identification of the subgroup of patients that may benefit from allogeneic stem cell transplantation may lead to transplantation of patients with less disease burden and a lower clinical risk score and therefore improve the chances of a favorable outcome after SCT.

## Data Availability

data is available upon reasonable request from the corresponding author.

## Abbreviations

MKD: Mevalonate Kinase Deficiency
SCT: Stem cell transplantation
HSCT: Hematopoietic stem cell transplantation
HIDS: Hyper Immunoglobulinemia D and periodic fever syndrome
MA: Mevalonic Aciduria
NSAIDs: Non-steroidal anti-inflammatory drugs
GvHD: Graft-versus-host-disease
EBMT: The European Society for Blood and Marrow Transplantation
HLH: Hemophagocytic lymphohystiocytosis
PBSC: Peripheral blood stem cells
MSD: Matched sibling donor
MUD: Matched unrelated donor
MMUD: Mismatched unrelated donor
MMRD: Mismatched related donors
PRES: Posterior reversible encephalopathy syndrome
CDA: Congenital dyserythropoietic anemia
DADA2: Deficiency of adenosine deaminase type 2 (
ALL: Acute lymphoblastic leukemia
CAPS: Cryopyrin-associated periodic syndrome
PAMI: PSTPIP1-associated myeloid-related proteinemia inflammatory syndrome
